# Endogenous and Exogenous Stimuli-Responsive Drug Delivery Systems for Programmed Site-Specific Release

**DOI:** 10.3390/molecules24061117

**Published:** 2019-03-21

**Authors:** Ali Raza, Tahir Rasheed, Faran Nabeel, Uzma Hayat, Muhammad Bilal, Hafiz M. N. Iqbal

**Affiliations:** 1 School of Biomedical Engineering, Shanghai Jiao Tong University, Shanghai 200240, China; aliraza86@sjtu.edu.cn (A.R.); uzmahayat@sjtu.edu.cn (U.H.); 2 School of Chemistry and Chemical Engineering, State Key Laboratory of Metal Matrix Composites, Shanghai Jiao Tong University, Shanghai 200240, China; masil@sjtu.edu.cn (T.R.); frnnbl@sjtu.edu.cn (F.N.); 3 School of Life Science and Food Engineering, Huaiyin Institute of Technology, Huaian 223003, China; 4 Tecnologico de Monterrey, School of Engineering and Sciences, Campus Monterrey, Ave. Eugenio Garza Sada 2501, Monterrey CP 64849, Mexico

**Keywords:** stimuli-responsive, polymeric carriers, endogenous, exogenous, drug delivery, nanotechnology, biomedical applications

## Abstract

In this study, we reviewed state-of-the-art endogenous-based and exogenous-based stimuli-responsive drug delivery systems (DDS) for programmed site-specific release to overcome the drawbacks of conventional therapeutic modalities. This particular work focuses on the smart chemistry and mechanism of action aspects of several types of stimuli-responsive polymeric carriers that play a crucial role in extracellular and intracellular sections of diseased tissues or cells. With ever increasing scientific knowledge and awareness, research is underway around the globe to design new types of stimuli (external/internal) responsive polymeric carriers for biotechnological applications at large and biomedical and/or pharmaceutical applications, in particular. Both external/internal and even dual/multi-responsive behavior of polymeric carriers is considered an essential element of engineering so-called ‘smart’ DDS, which controls the effective and efficient dose loading, sustained release, individual variability, and targeted permeability in a sophisticated manner. So far, an array of DDS has been proposed, developed, and implemented. For instance, redox, pH, temperature, photo/light, magnetic, ultrasound, and electrical responsive DDS and/or all in all dual/dual/multi-responsive DDS (combination or two or more from any of the above). Despite the massive advancement in DDS arena, there are still many challenging concerns that remain to be addressed to cover the research gap. In this context, herein, an effort has been made to highlight those concerning issues to cover up the literature gap. Thus, the emphasis was given to the drug release mechanism and applications of endogenous and exogenous based stimuli-responsive DDS in the clinical settings.

## 1. Introduction

**Figure 1:**
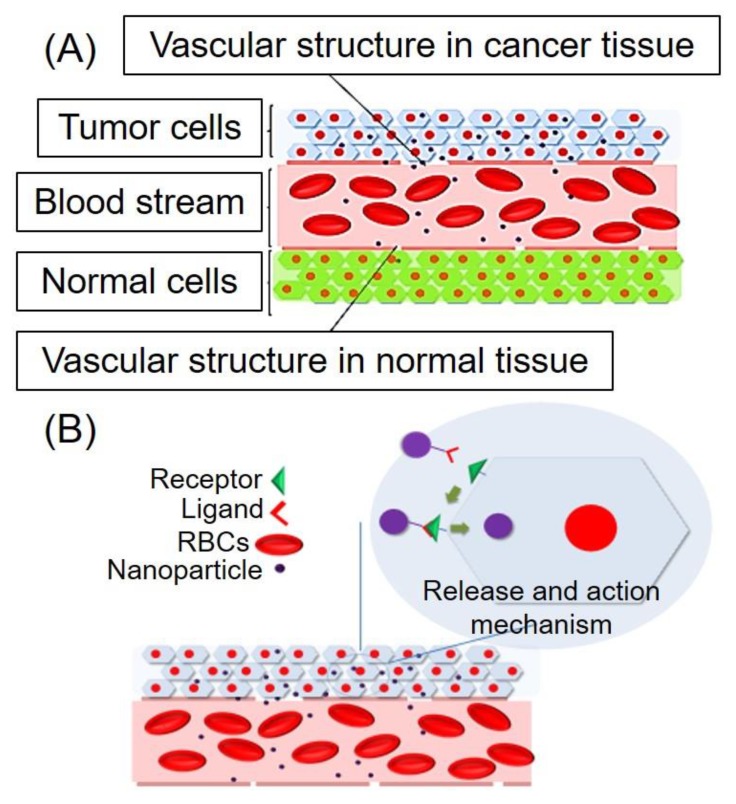
Schematic presentation of drug targeting by (**A**) enhanced permeability and retention (EPR) effect and (**B**) active targeting using tumor-targeted ligands.

Development of nanoparticle-based drug delivery systems is considered as a promising research area for pharmaceutical/biomedical researchers because of their potential of enhancing the efficacy of drugs particularly anti-cancer, antiviral, and antimicrobial agents [1]. Apart from drug delivery, nanoparticles have also been applied widely in biomedical imaging because of the potential of targeting enhancement, which allows incorporation of contrast agents and the ability to tune pharmacokinetic profile [2]. In addition, both abilities mentioned above of nanoparticles are found in theranostic nanoparticles, which offer simultaneous diagnosis, drug delivery for treatment, and monitoring of drug response [3]. Commonly employed nanoparticles for drug delivery are liposomes, polymeric nanoparticles, micelles, protein-based nanoparticles, and inorganic nanoparticles [4,5]. Enhancement of the efficacy of drugs by formulating nanoparticles drug delivery systems (NDDS) is mainly due to diseased tissue targeting ability without affecting healthy tissue [5]. Targeting the ability of nanoparticles was previously based on passive targeting, which involved enhanced permeability and retention effect due to poor vascular structure and lymphatic drainage of diseased tissue (tumor) [6,7] (Figure 1A). Later on, active targeting was achieved by ligands attachment on the surface of nanoparticles, which binds with specific cell targets present only in diseased tissue, which leads to cellular uptake of nanoparticles (Figure 1B). However, at present, most of the marketed NDDS is based on passive targeting [8]. These passive targeted nanocarriers have prolonged biological half-life, but premature drug release is a significant drawback.

Recently, NDDS with stimuli-responsive behavior have been reported and gained positive attention by researchers [9]. Two approaches can be adopted in designing stimuli-responsive DDS. In one approach, endogenous stimuli (internal stimuli/biological stimuli), which are mainly unique for diseased tissue, can be exploited for enhancing drug action specificity. These include pH and different levels of glutathione (GSH) concentration due to oxidative stress and specifically over-expressed enzymes [10]. It requires selection of appropriate material for designing nanocarriers, which respond to specific endogenous stimulus, leads to structure disruption of nanocarriers, which results in the abrupt release of the enclosed drug. In the second approach, physical stimuli are applied externally to targeted tissue after administration of drug-loaded specific nanocarriers. These exogenous stimuli include temperature, light, magnetic field, electric field, and ultrasound [1]. Application of these exogenous stimuli is responsible for alteration/disruption of the structure of specifically designed nanocarriers, which lead to drug release at targeted tissue [11,12]. Using stimuli-responsive drug delivery systems, the problem of premature drug release can be overcome [13]. Therefore, a research trend has been diverted toward stimuli-responsive DDS, and a combination of two or more stimuli-responsive systems has also been reported to increase targeting efficiency [11,14,15]. In this review, we discussed endogenous and exogenous stimuli along with dual/multi stimuli-responsive DDS in light of recently published reports. Furthermore, challenges in the clinical translation of these novel types of DDS are discussed.

## 2. Endogenous Stimuli-Responsive DDS

The endogenous stimulus of biochemical and chemical origin includes redox-responsive, enzyme responsive, pH-responsive, and ionic microenvironment responsive drug delivery systems. These DDS trigger the delivery of drugs by regulating the microenvironment tissues, over-expression of specific enzymes, antibody-antigen interaction, and recognition of host-guest moieties in a specific state.

### 2.1. Redox-Responsive DDS

**Figure 2:**
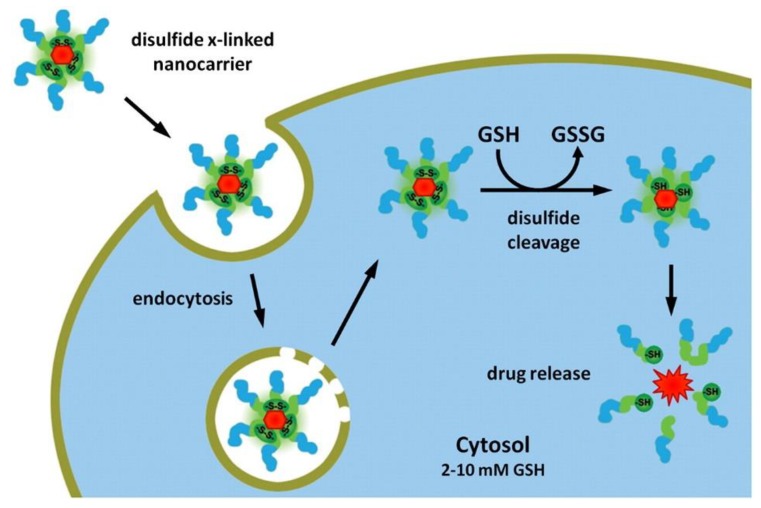
Schematic illustration of nanocarriers in the redox-responsive mechanism. The drug-carrying nanocarriers enter the cell through the endocytosis process. Glutathione (GSH) reduces the disulfide bonds after approaching the cytosol and subsequently erupts and releases the drug. Reprinted from Fleige et al. [18], with permission from Elsevier. Copyright (2012) Elsevier B.V.

Redox-responsive materials play a potential role in extracellular and intracellular sections of diseased tissues or cells for redox species with variable concentrations. The concentration of a reducing agent such as glutathione is two times greater in cellular cytosol and nucleus as compared to the intercellular (endosomes) and extracellular fluids. The redox potential prevailing between extracellular (oxidative) and intracellular (reductive) space is associated with extra-cellular and intracellular glutathione concentration [16]. During delivery of DNA or siRNA, it is essential that the payload is secured and is released inside the cell [17]. Given these generalizations, redox-sensitive nanocarriers, therefore, become more favorable for intracellular delivery particularly for gene delivery (Figure 2) [18]. Furthermore, increased concentration of the oxidizing agents (hydrogen peroxide and superoxide anions) is due to amplified cancer cells. However, redox-sensitive copolymers are desired for ex vivo applications in biomedical fields because of reversible nature and facile techniques for protein imitating to govern biosensing, cell culture, and diagnostics.

Since several therapeutics are active in the cell nucleus or cytosol, the development of drug delivery systems based on glutathione-responsive assemblies have attained more attention. Most of the glutathione-responsive entities are similar to design as of pH-responsive materials with acid-labile groups. Mostly, there exists a disulfide linkage in glutathione-responsive block copolymers between hydrophilic and hydrophobic blocks, which creates micelles formation also known as ‘shell-sheddable’. When these micelles come across the glutathione, they become destabilized and deliver the therapeutic material. Disulfide linkers in glutathione-responsive micelles have shown from in vivo and in vitro studies that they meet requirements of the drug carrier while in circulation, and are easily destabilized when entering the cells and releases the therapeutic materials [19]. Micelles which show a response to glutathione also make use of reducible linkers of thiols, which are attached to the shell or core of the micelles or use disulfide linkage to link polymer blocks to the drug molecules.

**Figure 3:**
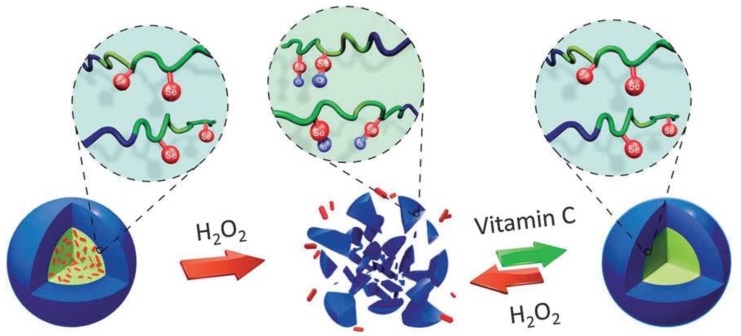
Schematic illustration showing redox-responsive assembly and disassembly of selenium (in red color) functionalized poly(ethylene glycol-poly(acrylic acid) (PEG-b-PAA) polymers [21]. Reprinted from Ren et al. [21], with permission from The Royal Society of Chemistry. Copyright (2012) The Royal Society of Chemistry.

Higher amounts of reducing materials in intracellular sections help the polymers with disulfide linkages whereas polymers containing hydrophobic blocks, i.e., poly-(propylene sulfide) are sensitive to the oxidative atmosphere of most of the diseases. The nanostructure disassembly occurs as a result of disturbance of hydrophilic/hydrophobic equilibrium in the solution assemblies from sulfone moieties and oxidation of sulfide entities to form hydrophilic sulfoxide [20]. A similar approach was used by Ren et al. in self-assembled nanostructures eruption by employing the oxidation-induced solubility variations from selenium block copolymers. The spherical micelles were prepared from copolymers in an aqueous medium with hydrophobic selenium cores. The oxidation of the selenium to selenoxide from hydrogen peroxide enhanced the hydrophilicity and disassembled the micelles (Figure 3) [21]. By adding reducing agents, spherical micelles were reproduced. The reversible formation of spherical micelles showed that these selenium-containing nano ampules were responsive under oxidizing and reducing conditions. Thereby, redox-responsive characteristics were fully retrieved [21].

A similar idea could be applied to polymersomes. For instance, Cerritelli et al. observed reductive disruption of poly(ethylene glycol)-disulfide poly (propylene sulfide) (PEG-PPS) embedded with the self-quenching quantity of the fluorescent guest calcein, and, after cleavage, the fluorescence intensity was enhanced due to dequenching [22]. Furthermore, this group also studied the destabilization of polymersomes of a triblock copolymer, i.e., poly(ethylene glycol)-poly(propylene sulfide)-poly(ethylene glycol) (PEG-PPS-PEG) upon oxidation. These polymersomes when treated with oxidizing agents such as glucose oxidase or hydrogen peroxide, the hydrophobic PSS block resulted in the rupture of vesicles via oxidation to more hydrophilic poly(sulfoxides) and poly(sulfones) [23]. A vesicle formed from layer-by-layer (LbL) assembly has been employed for active compounds delivery in which nanocarriers are produced by depositing polyelectrolytes alternatingly onto a template. Caruso group produced redox-responsive capsules using poly(*N*-vinyl pyrrolidone) (PVPON) and poly (methyl methacrylate) (PMAA) and a crosslinker of disulfide, which could deliver plasmid DNA and doxorubicin [24,25]. These doxorubicin-loaded capsules showed 5000-fold enhanced cytotoxicity when compared to doxorubicin only [24]. So far, the disulfide linkage has been used frequently for designing nanocarriers sensitive to reduction. The only concern in the early stages was in vivo stability due to the presence of cysteine and glutathione in the extracellular compartment, which could lead to early eruption [26]. This problem could be avoided by using manifold disulfide linkages, by adjusting the number of disulfide cross-links. The enhanced stability of the carriers, as well as lower transfection efficacy, are achieved due to the very high amount of cross-linkages [27,28].

### 2.2. pH-Responsive DDS

**Figure 4:**
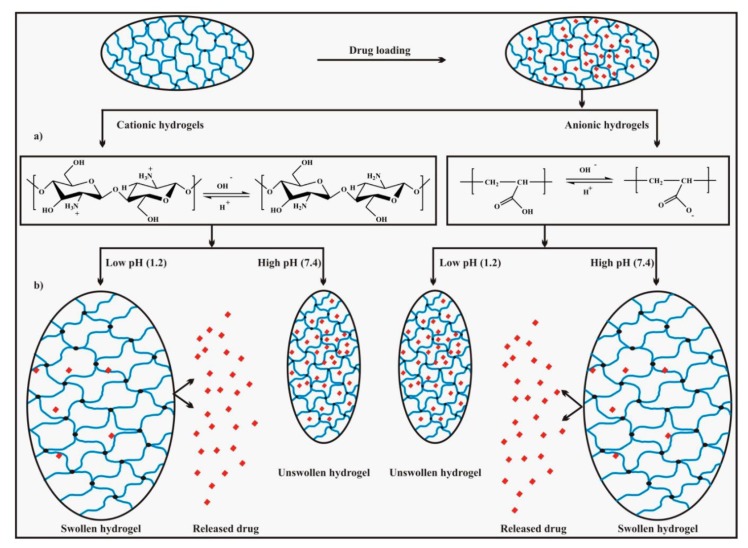
(**a**) pH-dependent ionization of specific acidic or basic functional groups on hydrogel chains responsible for swelling, (**b**) pH-dependent swelling, and drug release mechanism. Reproduced from Rizwan et al. [36], an open-access article distributed under the terms and conditions of the Creative Commons Attribution (CC BY) license (http://creativecommons.org/licenses/by/4.0/).

The pH-responsive DDS should be responsive and stable to slightly lower and physiological pH values (5.0–6.5, 7.4) for various biological applications. This design assists the therapeutics release inside the cell and surrounding tissues for the drug delivery purpose. This is because the endolysosomal sections are made upon internalization of several drug carriers. Additionally, the slight difference between the pH of tumor tissues versus normal tissue makes pH-responsiveness an ideal way to target the tumors with chemotherapeutics [29]. For other biological applications, such as bioseparations, biofiltration, and anti-biofouling, the ranges of pH for response versus stability are case-specific, with a key parameter being the use of protein-friendly conditions that maintain bioactivity [30]. Generally, two different strategies are being used to design the alternating copolymer-based pH-responsive self-assemblies. The first approach includes the incorporation of the acid-functionalized groups to the polymeric backbone. It may also be used to conjugate active drugs to the side chain of the polymer. These active drug groups may initiate the pH-response, which can accompany the conformational changes in all parts of the polymeric backbone. This change in conformation alters the nanostructures, which may result as self-assembly of the polymer. Following this pH-response phenomenon, several polymers, e.g., poly(lactic acid) (PLA), polycarbonates, poly(e-caprolactone) (PCL), acrylic acid, methacrylic acid (MAA), acrylonitrile (AN), polyketals, and polyanhydrides have been used to engineer pH-responsive DDS [31,32]. Consequently, the amphiphilic balance of the copolymer disrupts because of cleavage of the pH-triggered bond. This anomaly results in the breakage of the self-assembly or degrading of the nanocarrier and release of encapsulated therapeutics occur. The most striking nanocarriers for drug delivery purposes are degradable polymeric materials because these materials evade renal clearance [33]. On the other hands, the drugs that are covalently attached to the carriers, have numerous advantages including enhanced drug stability, improved circulation time, improved biodistribution, and condensed drug toxicity. For example, Lee and coworkers have used a double-hydrophilic hyperbranched copolymer poly(ethylene glycol-hb-glycerol) (PEG-hb-PG), which can self-assemble into micelles. In these constructs, the doxorubicin (an anticancer drug) was incorporated into the PG unit of the hyperbranched polymer through acid-labile hydrazone linkages. This attachment reduces the hydrophobic character of the PG unit and the resulting amphiphilic copolymer formed micelles in aqueous solutions. Upon breaking the formed hydrazone bond at a pH = 5.0 (in solution), or in the endolysosomal parts of the HeLa cells, the doxorubicin was released, and the micelles disassembled into relatively smaller PEG-hb-PG monomers. Therefore, the arrangement of hyperbranched structure with-pH-cleavable entities results in the formation of biocompatible polymer with improved drug loading capacity and enhanced efficient release [34]. Other important acid-cleavable structures that can be employed in copolymer assemblies include catechol, carbamate, and Schiff base [35,36]. The pH-responsive assemblies involve complete, or some part of the polymer, in triggering the stimuli-responsive alteration of the hydrophilic character, which results in the disruption of the self-assembling behavior of the nanostructure. pH-dependent swelling and drug release mechanism are shown in Figure 4 [36]. The hydrophilic changes that depend upon the pH have been widely used to activate the disassembling of the nanostructures into the monomers. For example, Manganiello and coworkers [37] have reported the disassembly of the micelles at endosomal pH, which results in the enhanced release of the cytoplasmic delivery of the nucleic acid. In another approach, Doncom’s group [38] benefiting from the pH-dependent protonation of amine groups presented a DDS that enhanced the hydrophilic character of an amphiphilic copolymer. This enhanced hydrophilic character leads to a pH-dependent transformation such as vesicle to micelle. Furthermore, the copolymer nanostructures can easily release a hydrophilic dye through the structural reorganization. These constructs can be applied as a pH-sensitive drug release system within tumor tissues or acidic intracellular portions. Sant et al. [39] utilize the (PEG-b-P(AlA-co-MAA) block copolymer for the oral application of a pH-sensitive micelle. The formed aggregates release the drug at a physiological value of pH. In this approach, the deprotonation of the carboxyl group results in the transformation of the hydrophobic core into hydrophilic, which disrupts the self-assembly and releases the guest molecule.

**Figure 5:**
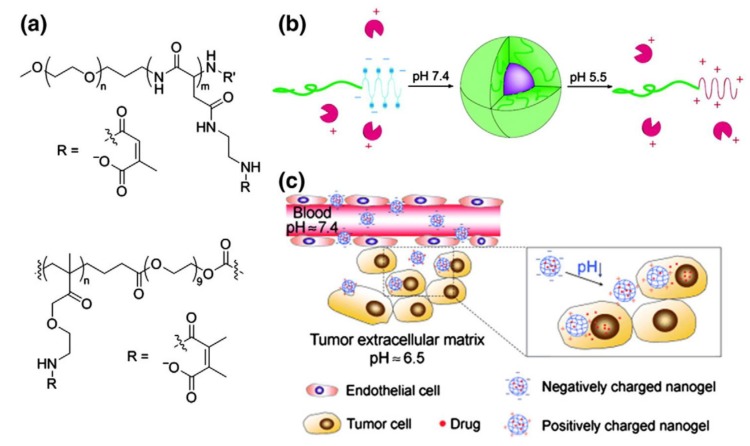
(**a**) Structure of a micellar charge-conversion nanocarrier (top) and a charge-conversion nanogel (bottom). (**b**) Formation and dissociation of a charge-conversion micelle encapsulating and releasing a positively-charged protein in dependence on the polymer charge. (**c**) Schematic illustration of the performance of the drug-loaded pH-responsive charge-conversion PAMA–DMMA nanogel. In the acidic tumor extracellular environment, the PAMA-DMMA nanogel is activated to be positively charged and is, thus, readily internalized by tumor cells with subsequent intracellular drug release. Reprinted from [44,45], with permission from John Wiley and Sons and American Chemical Society, respectively. Copyright (2010) WILEY-VCH Verlag GmbH & Co. KGaA, Weinheim and (2007) American Chemical Society.

Alternatively, the acid degradable units can also be used for the loading of the drug to the hydrophobic part of the amphiphilic polymer. Bae et al. used a block copolymer (PEG-b-PAsp) for the purpose. The doxorubicin drug was attached to the formed aggregates via a pH-responsive hydrazone bond [40,41]. Additionally, the micelle surface was functionalized with a folate ligand to increase the tumor-specific uptake. Aryal et al. [42] used a similar linking behavior to the PEG-b-PLLA micelle for the delivery of cisplatin. Both systems operate well at pH 6 or lower for the enhanced release of drugs. Likewise, Bae et al. prepared charge-conversion micelles for the delivery of proteins. The micelles were formed as a result of attachment of methyl maleate to the aspartate block of a PEG-pAsp, which carry a negative charge under physiological conditions. The methyl maleate group breaks as these micelles enter the cell. This cleavage results in free positive charge (amine) and the loaded protein are released at endosomal pH (Figure 5) [42,43,44,45].

### 2.3. Enzyme-Responsive DDS

**Figure 6:**
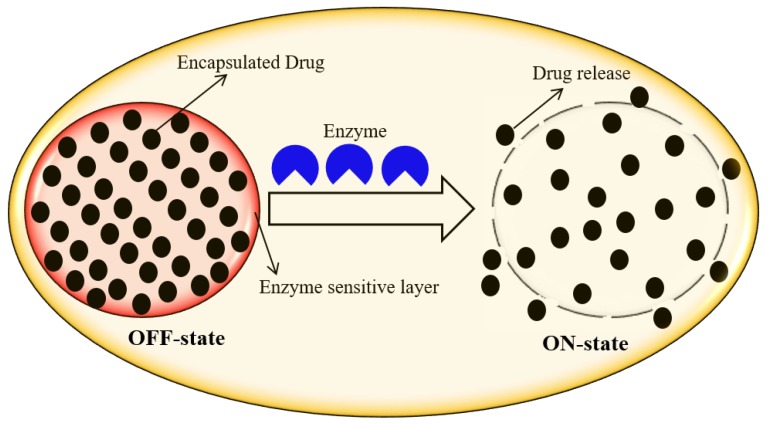
Enzyme-responsive drug delivery system. OFF-state: before enzyme action, and ON-state: after enzyme action. Reprinted from Rasheed et al. [1], with permission from Elsevier. Copyright (2018) Elsevier Ltd.

Enzyme-responsive DDS is a system that undergoes macroscopic transitions in its physicochemical properties upon the biocatalytic action of the enzyme [1]. Enzyme-based regulation and/or dysregulation in the intracellular microenvironment and their involvement in all biological and metabolic processes play a key role in designing tremendously promising responsive element for DDS. Enzyme-responsive DDS offer unique features such as biorecognition, process efficiency, sensitivity, selectivity, and catalytic efficacy, which are very advantageous in bio-nanomedicine [1,11]. Enzyme-oriented dysregulation in diseased cells/tissues also provoked new ultra-sensitive in-vivo DDS to identify and monitor different pathological states. In enzyme-responsive DDS, the drugs are released out of the drug-loaded carriers upon enzyme-assisted degradation of polymeric moiety. Figure 6 illustrates a simplified work mechanism of enzyme-responsive DDS [1]. Among different enzymes, proteases are of supreme interest to fabricate novel DDS, since they are often over-expressed in infectious diseases, such as cancer and inflammation. Trypsin (one of the most important digestive proteinases) plays a vital role in controlling the function of exocrine pancreatic secretion, which is involved in the stimulation of several other digestive enzymes [14]. Radhakrishnan et al. [15] fabricated dual enzyme-responsive hollow nanocapsules to deliver anticancer agents specifically inside cancer cells. The degradation of nanocapsule walls in the presence of trypsin or hyaluronidase lead to the release of the encapsulated drug molecules at a rapid rate. The integration of two or more enzymes synchronously, for enzyme-mediated DDS, could increase the accuracy and sensitivity of the method. Oxidoreductases have been considered as therapeutic targets due to their central role in oxidative stress and their involvement in diseases such as Alzheimer’s and cancer [12].

### 2.4. Ionic Microenvironment-Responsive DDS

Ionic microenvironment-responsive polymeric nanocarriers are being engineered by incorporating pendant acidic or basic functional entities to the polymer backbone, which strongly influence the degree of ionization. The drug release mechanism is subject to the degree of ionization of ionic microenvironment-responsive polymeric nano-carriers, which depend on the number of pendants acidic or basic groups. The high number of pendant acidic groups in the polymeric carriers caused an increased electrostatic repulsion between negatively charged carboxyl groups on different chains, which, in turn, results in greater swelling ratios at a high pH. On the other hand, polymeric carriers exhibited a high number of pendant basic groups, e.g., amines, can ionize and show electrostatic repulsion at a low pH [43]. Such functionalized polymeric nanocarriers triggered the loaded drug release either by accepting or donating the protons with response to pH and/or ionic strength changes in aqueous media. Generally, polymers with lower critical solution temperature (LCST) transitions are of supreme interest to develop ionic microenvironment-responsive DDS. For instance, PNIPAM, cellulose derivatives, and poly(vinyl ether), poly (*N*-vinyl caprolactam) are some examples that have been observed with LCST transitions in aqueous solution [46,47]. Zhang et al. [43] reported a fast and simple method for the preparation of novel pH- and ionic-strength-sensitive hydrogel membranes for drug delivery and tissue engineering applications. The hydrogels were formed by the intermolecular cross-linking of carboxymethyl dextran (CM-dextran) using 1-ethyl-(3-3-dimethylaminopropyl) carbodiimide hydrochloride (EDC) and *N*-hydroxysuccinimide (NHS). The obtained results confirmed the pH-dependent swelling phenomenon due to the pendant acidic groups, as mentioned earlier. However, the hydrogels’ permeability was reversible and subject to the pH and ionic strength changes. Earlier, Zhao and Moore [48] also developed ionic strength-responsive hydrogels patterning the elements inside microchannels by in-situ photopolymerization of mixtures containing water and surfactant. In another study, polyelectrolyte hydrogels based on methacrylic acid (MAA) and acrylonitrile (AN) monomers were prepared in the presence of a cross-linker, *N*,*N*-methylenebisacrylamide [31]. Various ionic strength fluids were tested that indicate the influence on the degree of swelling of the gels due to the osmotic pressure between freely-mobile ions within the gels and ions in buffer solutions.

## 3. Exogenous Stimuli-Responsive DDS

Unlike endogenous stimuli-responsive DDS, the exogenous one has the potential benefit of overcoming inter-patient variability since, in these systems, drug release is controlled by external factors, which can be controlled precisely [49]. Different external stimuli have been reported, which can be used to control drug releases such as temperature, magnetic field, light, electrical field, and ultrasound. In designing these DDS, thermo-responsive polymers have a significant role since most of these stimuli respond by heat generation. This increase in temperature can stimulate drug release through temperature-sensitive materials.

### 3.1. Temperature-Responsive DDS

**Figure 7:**
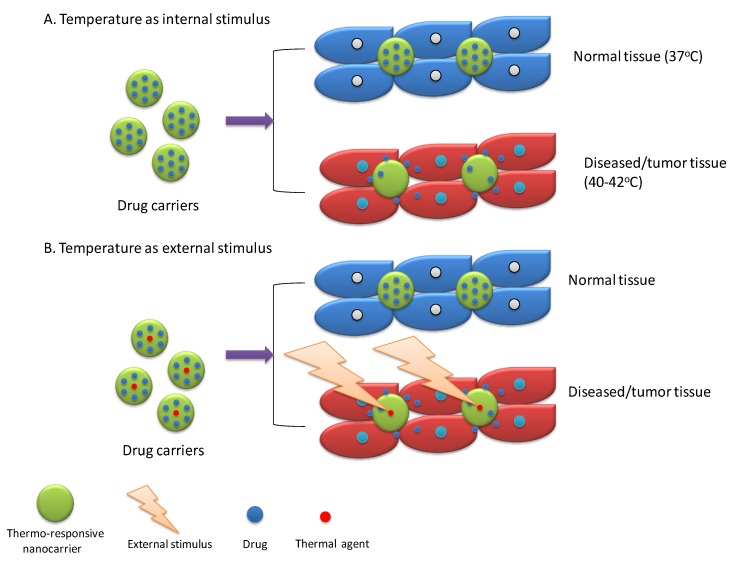
Schematic presentation of drug release activated by temperature responsive nanocarriers through temperature as an internal and external stimulus.

Temperature or thermo-responsive DDS are one of the widely explored exogenous stimuli-responsive DDS [9]. However, it can be used as an internal stimulus because diseased/tumor tissue usually have a higher temperature (~40–42 °C) as that of normal tissue (37 °C) [50]. Overall, thermo-responsive drug carriers retain drug in normal physiological temperature and release drug upon exposure of higher temperature of diseased tissue [13]. Two strategies have been reported for thermo-responsive drug release (Figure 7). First, thermo-responsive drug carriers are designed to respond to higher temperature for burst release of drug. This higher temperature is an intrinsic characteristic of diseased/tumor tissue (internal stimulus). For instance, Khoee and Karimi reported Polycaprolactone/(*N*-isopropylacrylamide) (PNIPAM) grafted graphene oxide nanoparticles loaded with 5-Fluorouracil and Quercetin for tumor treatment [51]. These dual drugs loaded NPs exhibited thermo-responsive behavior and a significant increase in drug release was reported at a higher temperature (40 °C) of diseased tissue as compared normal tissue temperature, i.e., 37 °C. Furthermore, in-vitro cytotoxicity studies showed a significant reduction in cell viability (~20%) at 40 °C as compared to that of 37 °C (~40%) when treated with dual drug loaded nano-particles [51]. In the second approach, drug carriers were designed for burst release in response to a higher temperature, which is achieved by external stimulus over targeted tissue. This external stimulus will stimulate a sensitive drug carrier component to produce heat, which, in turn, alters temperature-sensitive material present in carriers that lead to a burst release of drugs at the targeted site. This strategy is also usually used for inducing hyperthermia for thermal-based therapy along with chemotherapy. Commonly employed external stimuli for this purpose are a magnetic field, light, ultrasound, which are discussed in detail in forthcoming sections. For instance, Yang et al. [52] reported camptothecin and doxorubicin-loaded micelles composed of an amphiphilic diblock copolymer (P(HEMA-co-DMA)-b-P(AAm-co-AN)), a thermo-sensitive material, grafted with polypyrrole (PPy). PPy upon absorption of NIR produced heat due to photo-thermal effect and this increase in temperature, in turn, reported promoting drug release from micelles due to swelling of the thermo-sensitive polymer by hydrophobic to hydrophilic conversion. In-vivo studies in 4T1 tumor-bearing animal models also exhibited significant tumor reduction due to combined chemo and photo-thermal therapy [52]. Out of two strategies for thermo-responsive drug release, the external stimulus-based approach is promising for controlled delivery of drug to targeted tissue due to precise control and production feasibility, as compared to the internal stimulus-based approach, which is difficult to control due to a narrow temperature range.

Thermo-responsive polymers are the key member of these systems as they can respond to temperature change. There are two types of reported behaviors for thermo-responsive materials including lower critical solution temperature (LCST) and upper critical solution temperature (UCST) [20]. In the case of LCST, the decrease in temperature below LCST is responsible for increase swelling and vice versa due to the increase in hydrophilicity. In contrast, for polymers with UCST character, an increase in temperature above UCST leads to an increase in hydrophilicity. This alteration in hydrophilicity controls swelling behavior of drug carriers, which lead to drug release tuning [53]. For instance, PNIPAM, a promising building block for thermo-responsive DDS, has different solubility in water with temperature variability from its lower critical solution temperature (LCST), i.e., 32 °C. Above LCST, PNIPAM coils transform to globule which is water-insoluble and lead to controlling drug release due to the dominance of hydrophobic interactions [54]. Wu et al. [55] prepared mesoporous hydroxyapatite capped with PNIPAM and loaded with simvastatin to provide sustained delivery of simvastatin for the promotion of bone regeneration. Hydrophilic nature of PNIPAM below LCST was utilized for higher drug loading, and hydrophobic character above LCST was found useful for cell attachment and sustained release of simvastatin for up to 16 days [55]. Furthermore, their LCST can be modified by changing the ratio of hydrophilic and hydrophobic parts. This effect was used for controlling drug release by many researchers. For instance, Antoniraj et al. [56] synthesized poly (*N*-isopropylacrylamide)-g-carboxymethyl chitosan (PNIPAM-g-OCMC) copolymer-based doxorubicin-loaded polymeric nanoparticles using 1-(3-Dimethylaminopropyl)-3-ethylcarbodiimide/*N*-hydroxysuccinamide (EDC/NHS) as the coupling agent via acid-amine coupling reaction. Then, using PNIPAM-g-OCMC, doxorubicin-loaded polymeric nanoparticles (D-PNPs) were developed for thermo-responsive drug release [56].

### 3.2. Photo/Light-Responsive DDS

**Figure 8:**
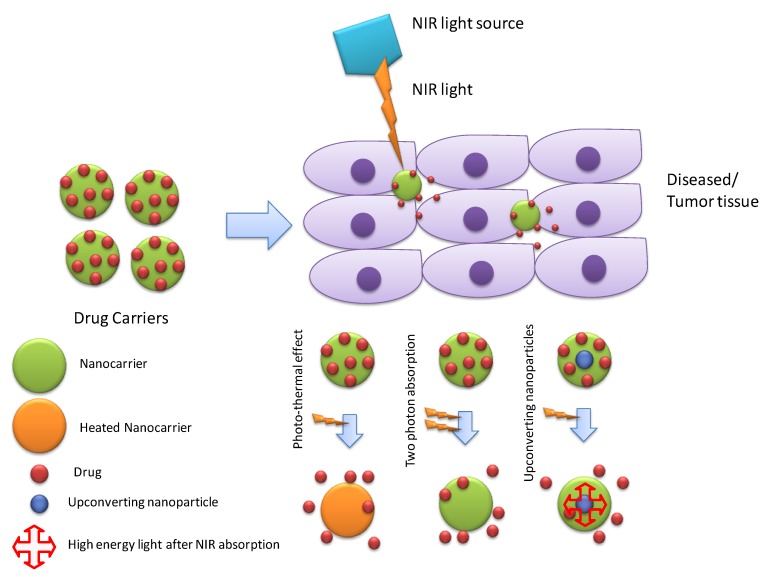
Mechanisms of drug release through NIR responsive DDSs.

Photo/light responsive DDS are widely explored systems by biomedical/pharmaceutical researchers to achieve targeted drug release. Light responsive systems have the potential benefit of better spatiotemporal control [57]. The different wavelength range of light (ultraviolet [58], visible [59], and near-infrared [60]) were reported to be used for controlling drug release. However, due to poor penetration, visible and UV light are not considered suitable for in vivo applications pertaining to therapeutics while the NIR range is the potential source of light for controlling drug release due to safety and better tissue penetration [61]. Three different mechanisms have been reported for drug release from NIR responsive systems: the photo-thermal effect, two-photon activation, and upconverting nanoparticles (Figure 8). The photo-thermal effect involves the conversion of light into heat by the photo-thermal agent, and this heat stimulates heat sensitive material, which disrupts the nanostructure, or its phase transition leads to fast drug release. Moreover, produced heat is also used for hyperthermic cytotoxicity against tumor tissue. Recently, Li et al. [62] reported multiple nanostructure lipid carriers encapsulated by liposomes loaded with hydrophilic drug AMD3100 and a hydrophobic NIR photo-thermal agent IR780. IR780 produced heat after absorbing NIR light, which destabilized the liposomal membrane leading to drug release. Furthermore, IR780 also induced cytotoxic hyperthermia for the synergistic effect with chemotherapy. In-vivo antitumor efficacy against 4T1-luc tumor-bearing BALB/c mice showed tumor ablation ability of prepared NIR responsive DDS [62].

Many UV light-sensitive materials have been reported, which can respond to UV light and release the enclosed drug. However, the limitation of UV light also restricted their biomedical applications. This problem can be solved by two-photon absorption and upconverting nanoparticles (UCNP). In two-photon absorption (TPA), two-photon of NIR range (equivalent to a single photon of UV) is absorbed by two-photon NIR sensitive/some UV sensitive material for desired response [63]. For instance, Guardado-Alvarez et al. [64] reported mesoporous silica nanoparticles, which is capped with a disulfide-linked β-cyclodextrin cap. A two-photon absorption based photo-transducer (*N*1-(4-((1E,3E)-4-(4-(dipropylamino)phenyl)buta-1,3-dien-1-yl)phenyl)-*N*1-propylethane-1,2-diamine) (2PNT) was used to provide an electron for reduction of disulfide linker. As a result of the reduction, the β-cyclodextrin cap was removed, which leads to release of enclosed cargo. The synthesized delivery system was reported to respond to both one photon (UV/408 nm) and two photons (NIR/800 nm) and, therefore, was considered as a suitable drug vehicle for photo-activated drug release ability. UCNP can convert NIR light to UV light, which is another approach to activate high energy light-sensitive materials by the NIR range light [65]. For instance, Xiang et al. [61] synthesized amphiphilic di-block copolymer (with UV sensitive hydrophobic layer, poly(4,5-dimethoxy-2-nitrobenzyl methacrylate), and outer hydrophilic layer, poly(methoxy polyethylene glycol monomethacrylate) coated up-converting nanoparticles (NaYF4:Yb/Tm@NaYF4). Upon NIR irradiation (908 nm), UCNPs produced UV light, which was absorbed by the UV-sensitive part of the di-block copolymer causing hydrophilic-hydrophobic imbalance. This imbalance disrupted micelle structure, which resulted in the release of the encapsulated drug (DOX) [61]. Apart from targeted drug release, some NIR responsive systems with the synergistic effect of photodynamic therapy involved a photosensitizer in producing reactive oxygen species (ROS) in addition to chemotherapy, which have also been reported [66,67].

### 3.3. Magnetic-Responsive DDS

**Figure 9:**
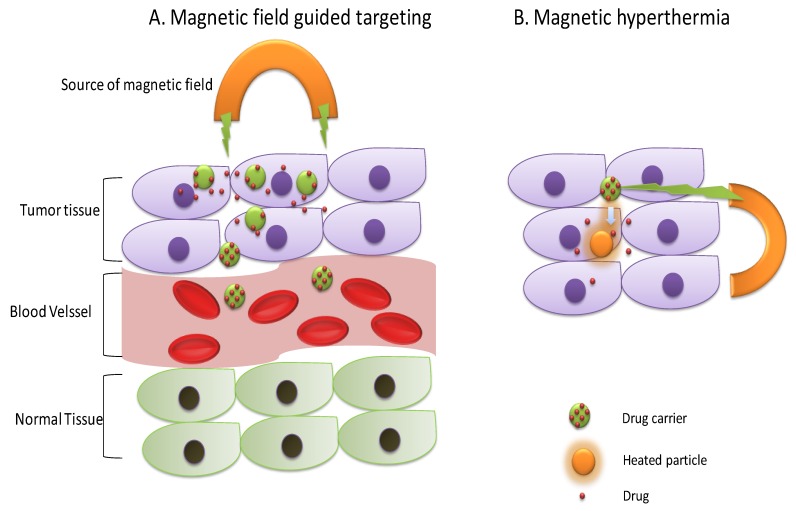
Mechanisms of drug release from magnetic field responsive DDS, which are guided by the magnetic field and magnetic hyperthermia.

The magnetic field can penetrate in the body tissue and is commonly employed for body imaging in MRI [68]. Apart from imaging, controlling drug release from magnetic field responsive carriers through external magnetic field stimulus have also been explored. Two mechanisms have been reported in this regard. The magnetic field induced hyperthermia for drug release [69] and the magnetic field guided drug targeting [70] (Figure 9). However, hyperthermia-based magnetic nanoparticles have been widely explored in the recent decade for drug delivery applications. In addition, local hyperthermia can also cause tumor inhibition along with the provision of imaging opportunity due to the presence of a magnetic response [71]. Thirunavukkarasu et al. [69] synthesized super-paramagnetic iron oxide (Fe_3_O_4_) nanoparticles (SPIONs) based multifunctional nanoparticles for a theranostic purpose. They loaded SPIONs and doxorubicin in a temperature-sensitive PLGA matrix, which was found to respond for heat produced by SPIONs upon magnetic field exposure and released DOX. In-vitro studies showed temperature sensitivity of PLGA matrix as ~39% drug release, which was observed at 37 °C and ~57% drug release was observed at 45 °C after 24 h at a pH of 7.4. Furthermore, applying an alternating magnetic field (AMF) at 4.4 kW was found to elevate the temperature of medium containing prepared nanoparticles by 5.2 °C (from 37 °C to 42.2 °C). In-vivo antitumor efficacy against CT26 tumor-bearing mice showed significant tumor inhibition effect of multifunctional nanoparticles as compared to DOX or SPIONs/PLGA alone indicating the synergistic effect of thermal and chemotherapy. In addition, the MR sensitivity of SPIONs also facilitated in-vivo MR imaging. In another report, Wang et al. [72] reported implantable magnetic chitosan hydrogel (MCH) loaded with hydrophobic (rifampicin) and hydrophilic drug (adriamycin). Implantable MCH was found to respond to external low frequency alternating magnetic field (LAMF) and released drug in a pulsatile manner without producing magnetic hyperthermia. This system was proposed to solve controlling drug release of the implantable hydrogel in the desired manner instead of the passive way and to prevent post-surgical infections [72].

### 3.4. Ultrasound-Responsive DDS

**Figure 10:**
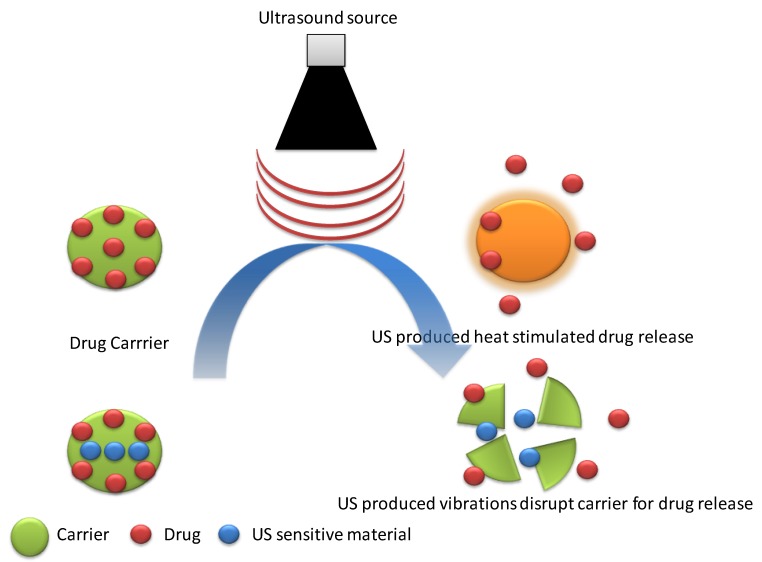
A schematic presentation of ultrasound responsive drug release through heat stimulation and US-induced vibrations.

Due to safety, tissue penetration, non-invasiveness, and better spatiotemporal control, ultrasound waves have been explored as an external stimulus on target-controlled drug release by many researchers [73]. Thermal, mechanical effects, and radiation forces produced by ultrasound waves are responsible for stimulating drug release for carriers (Figure 10) [74]. Paris et al. [75] reported mesoporous silica nanoparticles (MSN) PEGylated by the thermo-responsive linker (4,4′-azobis(4-cyanovaleric acid)). Applied external ultrasound (US) induced heat cleaved thermo-responsive linker leading to the removal of drug release protecting PEGylation and exposed positive charge on MSNs. Positively charged MSNs exhibited enhanced cellular uptake. Tepotecan-loaded US responsive MSNs showed a significant reduction in viability (~50%) of human osteosarcoma cells after 24 hours in the presence of ultrasound as compared to that without ultrasound exposure (~100%) [42]. In another report, Xin et al. [76] used PLGA nanoparticles to induce ultrasound responsive vibrations, which destabilized the membrane of liposomes enclosing PLGA NPs. Mitoxantrone-loaded US-sensitive liposomes showed enhanced drug release in the presence of US (~90%) as compared to that without US stimulus (~50%) after 10 hours. In addition, these liposomes exhibited prolonged blood half-life time, which makes them suitable for drug delivery applications [76].

### 3.5. Electrical-Responsive DDS

A weak electric field can be applied over targeted tissue after administration of electro-responsive drug carriers for controlled on-site drug release. Different mechanisms have been reported for controlling drug release through electrical stimulation including oxidation-reduction reaction [77], disruption structure of carriers [78], and stimulation of thermo-responsive carrier through electrically-produced heat [79]. Recently, Neumann et al. [80] reported a novel strategy for electro-responsive drug delivery. They utilized local pH change due to electrochemical reaction to control drug release through a pH-sensitive material. They synthesized drug loaded nanofilms using a pH-sensitive copolymer, poly(methyl methacrylate-co-methacrylic acid). Furthermore, they found that the pH change by the electrical signal was recovered quickly after the removal of stimulus due to buffer action, which prevented the OFF state drug release [80]. In another report, Xie et al. [48] reported electroresponsive polydopamine-polypyrrole microcapsules for on-demand drug delivery. Redox behavior of polypyrrole (PPy) was found to be responsible for electrically-stimulated drug release. Additionally, their prepared microcapsules were found to have higher drug loading, better biocompatibility, and cell adherence ability [81]. Table 1 summarizes all the above-discussed exogenous-based stimuli-responsive DDS.

## 4. Dual/Multi-Responsive DDS

Apart from single stimulus responsive DDS, some DDS have been reported that can respond to dual or multi-stimuli to enhance the efficiency of targeting [94]. These stimuli may be endogenous or exogenous or a combination of both. For instance, Zhang et al. [95] reported pH and redox (dual endogenous stimuli) responsive micelles for tumor targeting. They synthesized copolymers based on poly(ε-caprolactone) (PCL) and poly(ethylene glycol) (PEG) by disulfide (PCL-ss-PEG-ss-PCL) and acetal (PCL-acetal-PEG) linkages and their mixture was used to fabricate dual responsive micelles. Furthermore, micelles were functionalized with folic acid and loaded with Indocyanine G (ICG/photosensitizer) and Doxorubicin (DOX/chemotherapeutic agent). NIR light was used to sensitize ICG for thermal therapy. In-vivo tumor efficacy studies on BEL 7404 tumor-bearing nude mice showed irreversible tumor ablation due to the synergistic action of DOX (chemotherapy) and ICG (photo-thermal therapy) [95]. In another report, You et al. [96] reported ICG and cisplatin loaded NIR and redox-responsive (exogenous and endogenous stimuli) reactive smart nanoparticles for tumor targeting. In-vitro drug release studies showed a significantly higher drug release in the presence of both stimulus (99.35%) as compared to NIR alone (73.46%) or glutathione alone (58.45%) or without any stimulus (12.35%) after 72 h at a pH of 7.4. However, this difference was not remarkable in the case of pH 5.5 [96]. Hegazy et al. [97] reported a triple stimuli-responsive drug delivery system based on mesoporous silica and iron oxide nanoparticles. They used iron oxide (Fe_3_O_4_) nanoparticles as a core with mesoporous silica coating loaded with ICG and DOX and functionalized with thermoresponsive PNIPAAM through disulfide linker. Nanoparticles showed higher drug release at a higher temperature and in the presence of a reducing agent, tris(2-carboxyethyl) phosphine (TCEP) [97].

## 5. Concluding Remarks and Future Perspectives

In conclusion, the nano-tech aided engineering novel polymeric carriers are hugely growing in various types of drug delivery applications. With key scientific advances, massive research efforts are being made with a particular focus to construct DDS that can respond to the physiochemical-based surrounding changes that are either external or internal in a sophisticated manner with high-level control and precision. In this paper, we summarized the uniqueness of stimuli-responsive polymeric carriers and their potentialities for targeted drug delivery applications. Considering suitable examples, an array of stimuli-responsive DDS, for instance, redox-responsive, pH-responsive, temperature-responsive, photo/light-responsive, magnetic-responsive, ultrasound-responsive, and electrical-responsive DDS, and/or all-in-all dual/dual/multi-responsive DDS (combination or two or more from any of the above) have been discussed. For easy understanding and to highlight the work behavior of different DDS, schematic illustrations are also included by giving the focus on chemistry aspects, release behavior, and drug delivery applications.

Aside from the significant advancement in the biomedical/pharmaceutical at large and DDS arena, in particular, there are still many concerns that remain to be addressed. For instance, biocompatibility, biodegradability, non-toxicity, and safer elimination of the smart carriers from the biological system are some of the significant limiting factors that one needs to be considered prior to designing DDS. Furthermore, to induce fast responsiveness, the size of the carrier also plays a critical role. Nanosized carriers are being developed with comprisable mechanical characteristics, which is one of the important features required for biomedical-based applications. Besides processing drawbacks, administrative issues to get approval to use the designed polymeric-based smart DDS is another major bottleneck in the field. To avoid all these limitations, future investigations should be directed to introduce novel technologies to design DDS using polymeric materials with inherent properties such as biocompatibility, biodegradability, and non-toxicity. In conclusion, many polymer-based stimuli-responsive carriers offer substantial potential for biomedical applications. Thus, the external/internal stimuli-responsive carriers hold great promise for nanomedicine in the coming future.

## Figures and Tables

**Table 1:** Exogensous stimuli responsive DDS.

Stimulus	Delivery System	Structure	Drug-Loaded	Mechanism	Application	Reference
Temperature	Self-healable Hydrogel	P(NIPAM-FPA-DMA) co-polymer-based hydrogel with PEO_90_ dihydrazide as cross-linker.	Doxorubicin	High mobility of matrix	Targeted drug release, tissue engineering	[82]
	Nanogel	Chitosan grafted PNIPAM based nanogel assembly	Curcumin	Above LCST of PNIPAM coil to globule changes promoted drug release.	Targeted drug delivery	[83]
NIR light	Cancer cell membrane cloaked carrier-free nano-system	Doxorubicin/ICG nanoparticles encapsulated in the cracked cancer cell membrane	Doxorubicin/ICG	Photo-thermal based, thermal perturbation upon NIR irradiation.	Tumor ablation through synergistic photo-thermal and chemotherapy.	[84]
	MSNs based nanocarriers	Mesoporous silica nanoparticles (MSNs) coated with a lipid bilayer (DOPE-DOPC) and intermediate Polyethyleneimine layer.	Zoledronic acid and IR-780	Photochemical internalization mediated drug release.	Photodynamic and chemotherapy of the tumor.	[85]
	Nanoparticles	Hollow mesoporous Prussian blue nanoparticles filled with phase change material (1-tetradecanol) loaded with two drugs.	Doxorubicin and Camptothecin.	Melting of 1-tetradecanol resulted in the escape of 1-TD and drugs from the carrier.	Tumor treatment through synergistic photo-thermal and chemotherapy.	[86]
Magnetic field	Solid lipid nanoparticles	Magnetic nanoparticles coated with glyceryl monostearate and Pluronic F-68 loaded with Paclitaxel.	Paclitaxel	Magnetic hyperthermia, responsible for melting lipid layer, which promotes drug release.	Targeted drug delivery, Thermal therapy by magnetic hyperthermia	[87]
	Nanoparticles	Manganese ferrite (MnFe_2_O_4_) nanoparticles functionalized with mono/multilayers of chitosan and alginate sodium	Curcumin	Magnetic hyperthermia	Targeted drug delivery against the tumor, Imaging.	[88]
	Lipid-coated superparamagnetic nanoparticles	DPPC-DPPG coated iron oxides magnetic nanoparticles	Camptothecin	Magnetic hyperthermia	Targeted drug delivery for tumor treatment.	[89]
Ultrasound	Nanoparticle aggregates (NPA)	Drug-loaded PLGA nanoparticles were transformed into nanoparticles aggregates	Doxorubicin	Ultrasonic vibrations stimulated NPA dissociation promoting enhanced tumor uptake.	Targeted drug delivery to the disease site	[90]
	Microbubble	siRNA and poly(ethylene glycol)-b-poly(L-Lysine) (mPEG-b-PLLys) based cationic micelles encapsulated in phospholipid microbubble	si RNA	Enhanced permeability to tumor tissue by US exposure.	Image-guided tumor therapy.	[91]
Electric field	Nanocomposite film	Polypyrrole/graphene oxide nanocomposite film	Dexamethasone	Electrochemical reduction	On-demand drug delivery without the passive release of the drug.	[92]
	Hydrogel film	Acrylamide and N, N0-ethylene bisacrylamide polymerized hydrogel film with incorporated multi-walled carbon nanotubes.	Diclofenac sodium and ciprofloxacin	Electrostatic interactions variability	On-demand drug delivery	[93]

## References

[1] Rasheed T., Bilal M., Abu-Thabit N.Y., Iqbal H.M. (2018). The smart chemistry of stimuli-responsive polymeric carriers for target drug delivery applications. Stimuli Responsive Polymeric Nanocarriers for Drug Delivery Applications.

[2] Li C. (2014). A targeted approach to cancer imaging and therapy. Nat. Mater..

[3] Ahmed N., Fessi H., Elaissari A. (2012). Theranostic applications of nanoparticles in cancer. Drug Discov. Today.

[4] Bamrungsap S., Zhao Z., Chen T., Wang L., Li C., Fu T., Tan W. (2012). Nanotechnology in therapeutics: A focus on nanoparticles as a drug delivery system. Nanomedicine.

[5] Iqbal H.M., Keshavarz T. (2018). Bioinspired polymeric carriers for drug delivery applications. Stimuli Responsive Polymeric Nanocarriers for Drug Delivery Applications.

[6] Raza A., Hayat U., Rasheed T., Bilal M., Iqbal H.M. (2018). Redox-responsive nano-carriers as tumor-targeted drug delivery systems. Eur. J. Med. Chem..

[7] Bertrand N., Wu J., Xu X., Kamaly N., Farokhzad O.C. (2014). Cancer nanotechnology: The impact of passive and active targeting in the era of modern cancer biology. Adv. Drug Deliv. Rev..

[8] Bobo D., Robinson K.J., Islam J., Thurecht K.J., Corrie S.R. (2016). Nanoparticle-based medicines: A review of FDA-approved materials and clinical trials to date. Pharm. Res..

[9] Gu M., Wang X., Toh T.B., Chow E.K.H. (2018). Applications of stimuli-responsive nanoscale drug delivery systems in translational research. Drug Discov. Today.

[10] Li J., Ma Y.J., Wang Y., Chen B.Z., Guo X.D., Zhang C.Y. (2018). Dual redox/pH-responsive hybrid polymer-lipid composites: Synthesis, preparation, characterization and application in drug delivery with enhanced therapeutic efficacy. Chem. Eng. J..

[11] Hu X., Tian J., Liu T., Zhang G., Liu S. (2013). Photo-triggered release of caged camptothecin prodrugs from dually responsive shell cross-linked micelles. Macromolecules.

[12] Kundu J.K., Surh Y.J. (2010). Nrf2-Keap1 signaling as a potential target for chemoprevention of inflammation-associated carcinogenesis. Pharm. Res..

[13] Liu D., Yang F., Xiong F., Gu N. (2016). The smart drug delivery system and its clinical potential. Theranostics.

[14] Basel M.T., Shrestha T.B., Troyer D.L., Bossmann S.H. (2011). Protease-sensitive, polymer-caged liposomes: A method for making highly targeted liposomes using triggered release. ACS Nano.

[15] Radhakrishnan K., Tripathy J., Gnanadhas D.P., Chakravortty D., Raichur A.M. (2014). Dual enzyme responsive and targeted nanocapsules for intracellular delivery of anticancer agents. RSC Adv..

[16] Schafer F.Q., Buettner G.R. (2001). Redox environment of the cell as viewed through the redox state of the glutathione disulfide/glutathione couple. Free Radic. Biol. Med..

[17] Mintzer M.A., Simanek E.E. (2008). Nonviral vectors for gene delivery. Chem. Rev..

[18] Fleige E., Quadir M.A., Haag R. (2012). Stimuli-responsive polymeric nanocarriers for the controlled transport of active compounds: Concepts and applications. Adv. Drug Deliv. Rev..

[19] Cheng R., Feng F., Meng F., Deng C., Feijen J., Zhong Z. (2011). Glutathione-responsive nano-vehicles as a promising platform for targeted intracellular drug and gene delivery. J. Control. Release.

[20] Napoli A., Valentini M., Tirelli N., Müller M., Hubbell J.A. (2004). Oxidation-responsive polymeric vesicles. Nat. Mater..

[21] Ren H., Wu Y., Ma N., Xu H., Zhang X. (2012). Side-chain selenium-containing amphiphilic block copolymers: Redox-controlled self-assembly and disassembly. Soft Matter.

[22] Cerritelli S., Velluto D., Hubbell J.A. (2007). PEG-SS-PPS: Reduction-sensitive disulfide block copolymer vesicles for intracellular drug delivery. Biomacromolecules.

[23] Napoli A., Boerakker M.J., Tirelli N., Nolte R.J., Sommerdijk N.A., Hubbell J.A. (2004). Glucose-oxidase based self-destructing polymeric vesicles. Langmuir.

[24] Yan Y., Johnston A.P., Dodds S.J., Kamphuis M.M., Ferguson C., Parton R.G., Caruso F. (2010). Uptake and intracellular fate of disulfide-bonded polymer hydrogel capsules for doxorubicin delivery to colorectal cancer cells. ACS Nano.

[25] Ng S.L., Such G.K., Johnston A.P., Antequera-García G., Caruso F. (2011). Controlled release of DNA from poly (vinylpyrrolidone) capsules using cleavable linkers. Biomaterials.

[26] Thorpe P.E., Wallace P.M., Knowles P.P., Relf M.G., Brown A.N., Watson G.J., Blakey D.C. (1987). New coupling agents for the synthesis of immunotoxins containing a hindered disulfide bond with improved stability in vivo. Cancer Res..

[27] Miyata K., Kakizawa Y., Nishiyama N., Harada A., Yamasaki Y., Koyama H., Kataoka K. (2004). Block catiomer polyplexes with regulated densities of charge and disulfide cross-linking directed to enhance gene expression. J. Am. Chem. Soc..

[28] Oba M., Vachutinsky Y., Miyata K., Kano M.R., Ikeda S., Nishiyama N., Kataoka K. (2010). Antiangiogenic gene therapy of solid tumor by systemic injection of polyplex micelles loading plasmid DNA encoding soluble Flt-1. Mol. Pharm..

[29] Colson Y.L., Grinstaff M.W. (2012). Biologically responsive polymeric nanoparticles for drug delivery. Adv. Mater..

[30] Yu K., Han Y. (2009). Effect of block sequence and block length on the stimuli-responsive behavior of polyampholyte brushes: Hydrogen bonding and electrostatic interaction as the driving force for surface rearrangement. Soft Matter.

[31] Zhang K., Luo Y., Li Z. (2007). Synthesis and Characterization of a pH-and Ionic Strength-Responsive Hydrogel. Soft Mater..

[32] Chen W., Meng F., Cheng R., Zhong Z. (2010). pH-Sensitive degradable polymersomes for triggered release of anticancer drugs: A comparative study with micelles. J. Control. Release.

[33] Wang J., Byrne J.D., Napier M.E., DeSimone J.M. (2011). More effective nanomedicines through particle design. Small.

[34] Lee S., Saito K., Lee H.R., Lee M.J., Shibasaki Y., Oishi Y., Kim B.S. (2012). Hyperbranched double hydrophilic block copolymer micelles of poly (ethylene oxide) and polyglycerol for pH-responsive drug delivery. Biomacromolecules.

[35] Su J., Chen F., Cryns V.L., Messersmith P.B. (2011). Catechol polymers for pH-responsive, targeted drug delivery to cancer cells. J. Am. Chem. Soc..

[36] Rizwan M., Yahya R., Hassan A., Yar M., Azzahari A.D., Selvanathan V., Abouloula C.N. (2017). pH Sensitive Hydrogels in Drug Delivery: Brief History, Properties, Swelling, and Release Mechanism, Material Selection and Applications. Polymers.

[37] Manganiello M.J., Cheng C., Convertine A.J., Bryers J.D., Stayton P.S. (2012). Diblock copolymers with tunable pH transitions for gene delivery. Biomaterials.

[38] Doncom K.E., Hansell C.F., Theato P., O’Reilly R.K. (2012). pH-switchable polymer nanostructures for controlled release. Polym. Chem..

[39] Sant V.P., Smith D., Leroux J.C. (2004). Novel pH-sensitive supramolecular assemblies for oral delivery of poorly water soluble drugs: Preparation and characterization. J. Control. Release.

[40] Bae Y., Fukushima S., Harada A., Kataoka K. (2003). Design of environment-sensitive supramolecular assemblies for intracellular drug delivery: Polymeric micelles that are responsive to intracellular pH change. Angew. Chem..

[41] Bae Y., Kataoka K. (2009). Intelligent polymeric micelles from functional poly (ethylene glycol)-poly (amino acid) block copolymers. Adv. Drug Deliv. Rev..

[42] Aryal S., Hu C.M.J., Zhang L. (2009). Polymer− cisplatin conjugate nanoparticles for acid-responsive drug delivery. ACS Nano.

[43] Zhang R., Tang M., Bowyer A., Eisenthal R., Hubble J. (2005). A novel pH-and ionic-strength-sensitive carboxy methyl dextran hydrogel. Biomaterials.

[44] Du J.Z., Sun T.M., Song W.J., Wu J., Wang J. (2010). A tumor-acidity-activated charge-conversional nanogel as an intelligent vehicle for promoted tumoral-cell uptake and drug delivery. Angew. Chem..

[45] Lee Y., Fukushima S., Bae Y., Hiki S., Ishii T., Kataoka K. (2007). A protein nanocarrier from charge-conversion polymer in response to endosomal pH. J. Am. Chem. Soc..

[46] Furyk S., Zhang Y., Ortiz-Acosta D., Cremer P.S., Bergbreiter D.E. (2006). Effects of end group polarity and molecular weight on the lower critical solution temperature of poly (*N*-isopropylacrylamide). J. Polym. Sci. Part A Polym. Chem..

[47] Karewicz A., Zasada K., Szczubiałka K., Zapotoczny S., Lach R., Nowakowska M. (2010). “Smart” alginate–hydroxypropylcellulose microbeads for controlled release of heparin. Int. J. Pharm..

[48] Zhao B., Moore J.S. (2001). Fast pH-and ionic strength-responsive hydrogels in microchannels. Langmuir.

[49] Raza A., Hayat U., Rasheed T., Bilal M., Iqbal H.M. (2018). “Smart” materials-based near-infrared light-responsive drug delivery systems for cancer treatment: A review. J. Mater. Res. Technol..

[50] Liu M., Du H., Zhang W., Zhai G. (2017). Internal stimuli-responsive nanocarriers for drug delivery: Design strategies and applications. Mater. Sci. Eng. C.

[51] Khoee S., Karimi M.R. (2018). Dual-drug loaded Janus graphene oxide-based thermoresponsive nanoparticles for targeted therapy. Polymer.

[52] Yang J., Zhai S., Qin H., Yan H., Xing D., Hu X. (2018). NIR-controlled morphology transformation and pulsatile drug delivery based on multifunctional phototheranostic nanoparticles for photoacoustic imaging-guided photothermal-chemotherapy. Biomaterials.

[53] Karimi M., Zangabad P.S., Ghasemi A., Hamblin M.R. (2015). Smart Internal Stimulus-Responsive Nanocarriers for Drug and Gene Delivery.

[54] Park Y., Hashimoto C., Ozaki Y., Jung Y.M. (2016). Understanding the phase transition of linear poly (*N*-isopropylacrylamide) gel under the heating and cooling processes. J. Mol. Struct..

[55] Wu T., Tan L., Cheng N., Yan Q., Zhang Y.F., Liu C.J., Shi B. (2016). PNIPAAM modified mesoporous hydroxyapatite for sustained osteogenic drug release and promoting cell attachment. Mater. Sci. Eng. C.

[56] Antoniraj M.G., Kumar C.S., Kandasamy R. (2016). Synthesis and characterization of poly (*N*-isopropylacrylamide)-g-carboxymethyl chitosan copolymer-based doxorubicin-loaded polymeric nanoparticles for thermoresponsive drug release. Colloid Polym. Sci..

[57] Lino M.M., Ferreira L. (2018). Light-triggerable formulations for the intracellular controlled release of biomolecules. Drug Discov. Today.

[58] Brown A.A., Azzaroni O., Huck W.T.S. (2009). Photoresponsive Polymer Brushes for Hydrophilic Patterning. Langmuir.

[59] Hossion A.M., Bio M., Nkepang G., Awuah S.G., You Y. (2012). Visible light controlled release of anticancer drug through double activation of prodrug. ACS Med. Chem. Lett..

[60] Liu C., Zhang Y., Liu M., Chen Z., Lin Y., Li W., Qu X. (2017). A NIR-controlled cage mimicking system for hydrophobic drug mediated cancer therapy. Biomaterials.

[61] Xiang J., Tong X., Shi F., Yan Q., Yu B., Zhao Y. (2018). Near-infrared light-triggered drug release from UV-responsive diblock copolymer-coated upconversion nanoparticles with high monodispersity. J. Mater. Chem. B.

[62] Li H., Yang X., Zhou Z., Wang K., Li C., Qiao H., Sun M. (2017). Near-infrared light-triggered drug release from a multiple lipid carrier complex using an all-in-one strategy. J. Control. Release.

[63] Yang G., Liu J., Wu Y., Feng L., Liu Z. (2016). Near-infrared-light responsive nanoscale drug delivery systems for cancer treatment. Coord. Chem. Rev..

[64] Guardado-Alvarez T.M., Devi L.S., Vabre J.M., Pecorelli T.A., Schwartz B.J., Durand J.O., Zink J.I. (2014). Photo-redox activated drug delivery systems operating under two photon excitation in the near-IR. Nanoscale.

[65] Gwon K., Jo E.J., Sahu A., Lee J.Y., Kim M.G., Tae G. (2018). Improved near infrared-mediated hydrogel formation using diacrylated Pluronic F127-coated upconversion nanoparticles. Mater. Sci. Eng. C.

[66] Li Q., Li W., Di H., Luo L., Zhu C., Yang J., You J. (2018). A photosensitive liposome with NIR light triggered doxorubicin release as a combined photodynamic-chemo therapy system. J. Control. Release.

[67] Liu Y., Zhi X., Yang M., Zhang J., Lin L., Zhao X., Alfranca G. (2017). Tumor-triggered drug release from calcium carbonate-encapsulated gold nanostars for near-infrared photodynamic/photothermal combination antitumor therapy. Theranostics.

[68] Wang Y., Kohane D.S. (2017). External triggering and triggered targeting strategies for drug delivery. Nat. Rev. Mater..

[69] Thirunavukkarasu G.K., Cherukula K., Lee H., Jeong Y.Y., Park I.K., Lee J.Y. (2018). Magnetic field-inducible drug-eluting nanoparticles for image-guided thermo-chemotherapy. Biomaterials.

[70] Schleich N., Danhier F., Préat V. (2015). Iron oxide-loaded nanotheranostics: Major obstacles to in vivo studies and clinical translation. J. Control. Release.

[71] Zhou X., Wang L., Xu Y., Du W., Cai X., Wang F., Zheng Y. (2018). A pH and magnetic dual-response hydrogel for synergistic chemo-magnetic hyperthermia tumor therapy. RSC Adv..

[72] Wang Y., Li B., Xu F., Han Z., Wei D., Jia D., Zhou Y. (2018). Tough Magnetic Chitosan Hydrogel Nanocomposites for Remotely Stimulated Drug Release. Biomacromolecules.

[73] Paris J.L., Cabañas M.V., Manzano M., Vallet-Regí M. (2015). Polymer-grafted mesoporous silica nanoparticles as ultrasound-responsive drug carriers. ACS Nano.

[74] Luo Z., Jin K., Pang Q., Shen S., Yan Z., Jiang T., Jiang X. (2017). On-demand drug release from dual-targeting small nanoparticles triggered by high-intensity focused ultrasound enhanced glioblastoma-targeting therapy. ACS Appl. Mater. Interfaces.

[75] Paris J.L., Manzano M., Cabañas M.V., Vallet-Regí M. (2018). Mesoporous silica nanoparticles engineered for ultrasound-induced uptake by cancer cells. Nanoscale.

[76] Xin Y., Qi Q., Mao Z., Zhan X. (2017). PLGA nanoparticles introduction into mitoxantrone-loaded ultrasound-responsive liposomes: In vitro and in vivo investigations. Int. J. Pharm..

[77] Jeon G., Yang S.Y., Byun J., Kim J.K. (2011). Electrically actuatable smart nanoporous membrane for pulsatile drug release. Nano Lett..

[78] Servant A., Bussy C., Al-Jamal K., Kostarelos K. (2013). Design, engineering and structural integrity of electro-responsive carbon nanotube-based hydrogels for pulsatile drug release. J. Mater. Chem. B.

[79] Ge J., Neofytou E., Cahill T.J., Beygui R.E., Zare R.N. (2011). Drug release from electric-field-responsive nanoparticles. Acs Nano.

[80] Hosseini-Nassab N., Samanta D., Abdolazimi Y., Annes J.P., Zare R.N. (2017). Electrically controlled release of insulin using polypyrrole nanoparticles. Nanoscale.

[81] Xie C., Li P., Han L., Wang Z., Zhou T., Deng W., Lu X. (2017). Electroresponsive and cell-affinitive polydopamine/polypyrrole composite microcapsules with a dual-function of on-demand drug delivery and cell stimulation for electrical therapy. NPG Asia Mater..

[82] An H., Xu K., Chang L., Wang Y., Qin J., Li W. (2018). Thermo-responsive self-healable hydrogels with extremely mild base degradability and bio-compatibility. Polymer.

[83] Luckanagul J.A., Pitakchatwong C., Bhuket P.R.N., Muangnoi C., Rojsitthisak P., Chirachanchai S., Rojsitthisak P. (2018). Chitosan-based polymer hybrids for thermo-responsive nanogel delivery of curcumin. Carbohydr. Polym..

[84] Zhang N., Li M., Sun X., Jia H., Liu W. (2018). NIR-responsive cancer cytomembrane-cloaked carrier-free nanosystems for highly efficient and self-targeted tumor drug delivery. Biomaterials.

[85] Liu J., Karaman D.Ş., Zhang J., Rosenholm J.M., Guo X., Cai K. (2017). NIR light-activated dual-modality cancer therapy mediated by photochemical internalization of porous nanocarriers with tethered lipid bilayers. J. Mater. Chem. B.

[86] Chen H., Ma Y., Wang X., Zha Z. (2017). Multifunctional phase-change hollow mesoporous Prussian blue nanoparticles as a NIR light responsive drug co-delivery system to overcome cancer therapeutic resistance. J. Mater. Chem. B.

[87] Oliveira R.R., Carrião M.S., Pacheco M.T., Branquinho L.C., de Souza A.L.R., Bakuzis A.F., Lima E.M. (2018). Triggered release of paclitaxel from magnetic solid lipid nanoparticles by magnetic hyperthermia. Mater. Sci. Eng. C.

[88] Jardim K.V., Palomec-Garfias A.F., Andrade B.Y.G., Chaker J.A., Báo S.N., Márquez-Beltrán C., Sousa M.H. (2018). Novel magneto-responsive nanoplatforms based on MnFe2O4 nanoparticles layer-by-layer functionalized with chitosan and sodium alginate for magnetic controlled release of curcumin. Mater. Sci. Eng. C.

[89] Allam A.A., Potter S.J., Bud’ko S.L., Shi D., Mohamed D.F., Habib F.S., Pauletti G.M. (2018). Lipid-coated superparamagnetic nanoparticles for thermoresponsive cancer treatment. Int. J. Pharm..

[90] Papa A.L., Korin N., Kanapathipillai M., Mammoto A., Mammoto T., Jiang A., Cuneo G. (2017). Ultrasound-sensitive nanoparticle aggregates for targeted drug delivery. Biomaterials.

[91] Wang P., Yin T., Li J., Zheng B., Wang X., Wang Y., Shuai X. (2016). Ultrasound-responsive microbubbles for sonography-guided siRNA delivery. Nanomed. Nanotechnol. Biol. Med..

[92] Weaver C.L., LaRosa J.M., Luo X., Cui X.T. (2014). Electrically controlled drug delivery from graphene oxide nanocomposite films. ACS Nano.

[93] Curcio M., Spizzirri U.G., Cirillo G., Vittorio O., Picci N., Nicoletta F.P., Hampel S. (2015). On demand delivery of ionic drugs from electro-responsive CNT hybrid films. RSC Adv..

[94] An X., Zhu A., Luo H., Ke H., Chen H., Zhao Y. (2016). Rational design of multi-stimuli-responsive nanoparticles for precise cancer therapy. ACS Nano.

[95] Zhang L., Qin Y., Zhang Z., Fan F., Huang C., Lu L., Wang C. (2018). Dual pH/reduction-responsive hybrid polymeric micelles for targeted chemo-photothermal combination therapy. Acta Biomater..

[96] You C., Wu H., Wang M., Gao Z., Sun B., Zhang X. (2018). Synthesis and biological evaluation of redox/NIR dual stimulus-responsive polymeric nanoparticles for targeted delivery of cisplatin. Mater. Sci. Eng. C.

[97] Hegazy M., Zhou P., Wu G., Wang L., Rahoui N., Taloub N., Huang Y. (2017). Construction of polymer coated core–shell magnetic mesoporous silica nanoparticles with triple responsive drug delivery. Polym. Chem..

